# Assessment of Malondialdehyde and Organochlorine Pesticides in Aplastic Anemia Severity Groups: Insights Into Oxidative Stress and Exposure

**DOI:** 10.7759/cureus.59698

**Published:** 2024-05-05

**Authors:** Charu Goel, Nidhish Kumar, Anil Tripathi, Sunita Tiwari, Ashutosh Shrivastava

**Affiliations:** 1 Physiology, King George's Medical University, Lucknow, IND; 2 Physiology, Kalyan Singh Government Medical College, Bulandshahr, IND; 3 Pathology, Autonomous State Medical College, Shahjahanpur, IND; 4 Clinical Hematology, King George's Medical University, Lucknow, IND; 5 Hematology, Hind Institute of Medical Sciences, Barabanki, IND; 6 Physiology, Dr. Ram Manohar Lohia Institute of Medical Sciences, Lucknow, IND; 7 Centre for Advanced Research, King George's Medical University, Lucknow, IND

**Keywords:** heptachlor, delta-hch, thiobarbituric acid reactive substance, gc-ms/ms, pesticide, malondialdehyde, organochlorine, aplastic anemia

## Abstract

Background

There is little evidence that pesticide exposure is the primary cause of acquired aplastic anemia (AAA), even though the prevalence of aplastic anemia (AA) is substantially higher in underdeveloped countries than in affluent countries. AA caused by pesticides has not yet been fully understood. This study aimed to examine the potential link between plasma levels of malondialdehyde (MDA) and organochlorine pesticides (OCPs) as risk factors for developing AAA in the North Indian population.

Methods

This case-control study was conducted at a tertiary care hospital in North India. A total of 99 participants were chosen for the study, of whom 45 were cases of AA. These cases attended the Clinical Hematology department over a period of 1.5 years (May 2018 to November 2019). Forty-five controls were age and sex-matched, apparently healthy subjects. Written informed consent was obtained from each subject before performing the study. Exclusion criteria included patients unwilling to give consent, those using medication to treat AA, those genetically predisposed to AA, those with characteristics including granuloma and dysplasia of bone marrow, any other systemic illness, and subjects with a history of smoking, drinking, or using tobacco in any form. Gas chromatography-tandem mass spectrometry (GC-MS/MS) was used to evaluate the plasma levels of organochlorines. The estimation of plasma MDA, i.e., the lipid peroxide content, was measured.

Results

The severity of AA is significantly associated with plasma levels of α-Hexachlorocyclohexane (p = 0.040), Heptachlor (p = 0.006), Aldrin (p < 0.001), p,p'-Dichlorodiphenyldichloroethane (p = 0.004), Endosulfan sulfate (p = 0.010), and Methoxychlor (p = 0.001). There was a statistically non-significant difference in MDA levels between cases and controls (p = 0.145); however, a statistically significant linear increase in MDA levels (p < 0.001) was observed according to the severity of AA.

Conclusion

Our study suggests that oxidative stress may be linked to the severity of AA. Pesticide exposure (plasma organochlorine levels) could act as a stressor, potentially initiating an alarmin response of oxidative stress in the form of lipid peroxidation (MDA) from damaged tissue, which could then lead to suppression of hematopoiesis and be a possible factor in the development of AA.

## Introduction

Worldwide, 2-7 million people suffer from aplastic anemia (AA) [1]. It is a condition in which hematopoietic stem cells are replaced by adipose tissue, leading to a deficiency of RBCs, neutrophils, monocytes, and platelets in the blood [2]. Asian citizens are affected two to three times more often than those in other regions [3].

Pesticide exposure, especially to chlorinated hydrocarbons and organophosphate pesticides, has been associated with the development of AA [4]. The most commonly used pesticides include dichlorodiphenyltrichloroethane (DDT), lindane, and chlordane. Pentachlorophenol (PCP), a potentially dangerous chlorinated hydrocarbon used as a wood preservative, is produced when lindane is partially decomposed. PCP has been associated with AA and other blood disorders [5]. Potential mechanisms for acquired marrow failure include the susceptibility of hematopoietic stem cells to the lethal effects of hazardous chemical exposure, deterioration of hematopoietic cells due to the absence or impaired production and release of essential hematopoietic growth factors, a dysfunctional stromal milieu of the bone marrow, and cellular or humoral immune factors that suppress hematopoietic stem cells. Therefore, it appears that the diminished production of blood cells in AA may arise from either a gradual toxic impact on primitive hematopoietic cells over time or an immunological suppression of hematopoietic progenitor cells. According to one study, autoreactive T lymphocytes reduce hematopoiesis [6]. Numerous cases of AA with unknown etiologies may be caused by pesticide exposure. In one investigation, three people with AA were found to have been previously exposed to organochlorine pesticides [5]. Another study discovered that organophosphorus pesticides may increase the likelihood of developing AA [7]. Insecticides impair normal hematopoiesis and destroy hematopoietic stem cell and stromal cell populations on a quantitative and qualitative level, which ultimately results in severe bone marrow failure [8].

Case reports demonstrate a strong link between exposure to OCP and the subsequent onset of AA [5]. Pesticide use has been linked to DNA damage in pilots who participated in pesticide spraying, according to a study [9]. Another study showed that the reaction to breathing in paint fumes led to reversible liver, bone marrow, and nervous system poisoning [10]. According to research conducted in Lucknow, there is no significant correlation between children's exposure to organochlorine pesticides and AA [11].

Research unequivocally demonstrates that prolonged pesticide application by pesticide applicators leads to a buildup of pesticide residues in the blood, a reduction in antioxidant status, and a modification of the immune system that impacts humoral and cellular immunological responses, causing significant oxidative damage. Therefore, employees exposed to pesticides exhibit these changes more markedly and visibly than those exposed to fungicides [12].

Pesticides such as organochlorines, carbamates, and pyrethroids can result in oxidative stress by generating free radicals (FRs) and altering oxidative defense mechanisms. FRs production results in cellular damage, primarily through the lipid peroxidation of cell membranes, which is crucial for understanding the cellular toxicity of pesticides. Cell death may occur as a result of this process. A characteristic by-product of polyunsaturated fatty acid (PUFA) oxidation, which is caused by highly elevated levels of lipid peroxidation brought on by reactive oxygen species, is malondialdehyde (MDA). Because lipid peroxidation damages cell membranes, making them highly permeable and losing their integrity, it can disrupt the normal functioning of cells. Lipid hydroperoxides and MDA are the two main by-products of the breakdown of cell membranes. Thus, MDA levels can be used as an effective biomarker of oxidative stress [13].

Although developing countries have a significantly higher prevalence of AA than industrialized nations, there is very little evidence that pesticide exposure is the primary cause of idiopathic AA. This study aims to determine the relationship between plasma levels of organochlorines, oxidative stress biomarkers (MDA), and the risk of developing AA. Any chemical species capable of removing a hydrogen atom from the side chain of a PUFA, typically found in cell membranes, can initiate lipid peroxidation. The membranes of cells, the brain, muscles, and the liver all contain arachidonic acid, a polyunsaturated omega-6 fatty acid with several methylene double bonds, which FRs use as a source of hydrogen atoms. Arachidonic acid stimulates platelets to produce significant amounts of MDA. Vinblastine inhibits the conversion of arachidonate to MDA mediated by human platelet microsomes [14]. Aspirin or Indomethacin can also reduce MDA production by platelets [15].

PUFAs give rise to unstable lipid peroxides that readily degrade to form various compounds, including MDA. MDA, the primary metabolite of arachidonic acid, serves as a reliable biomarker for oxidative stress. MDA is a three-carbon dialdehyde formed during the metabolism of arachidonic acid and PUFAs; it is mutagenic, tumorigenic, and highly reactive. When prostaglandin endoperoxide (PGH2) is converted to 12-hydroxyheptadecatrienoate (HHT), MDA is also produced. It has a boiling point of 108°C and the chemical formula C_3_H_4_O_2_, with a 72.06 g/mol molar mass. The melting point ranges from 72 to 74°C. Generally, MDA appears as solid, needle-like crystals [16].

Several disorders are largely caused by oxidative stress, but conclusive evidence has been lacking. Due to issues with sensitivity, specificity, or invasive methods, in vitro markers of oxidative stress are ineffective in vivo. Direct detection techniques are not yet practical for clinical research. The status of enzyme- and non-enzyme-based antioxidants and damage products like lipid peroxidation, such as MDA and protein oxidation, can only be indirectly estimated. Protein carbonylation, reduced glutathione (GSH), total thiol (T-SH), and melatonin have been studied as indicators of enzymatic/non-enzymatic antioxidant status [17, 18].

A study of oxidative stress indices found higher levels of MDA in OP applicators compared to farmers, which may indicate increased lipid peroxidation in OP applicators [19]. Occupational health risks among male agricultural laborers aged 20-57 years, who mixed, prepared, and sprayed pesticides manually in Mango plantations at Malihabad, Rahimabad, and Mall, near Lucknow, were investigated. The study's findings provide strong evidence that the population exposed to pesticides had higher serum levels of MDA compared to the control group. Significant increases in MDA activity were associated with substantial declines in GSH enzyme activity (p < 0.001). The reduced antioxidant activity caused by exposure to pesticide mixtures resulted in increased peroxidation of red cell membranes in this study, leading to elevated levels of thiobarbituric acid reactive substances (TBARS). Many toxic OP pesticides attack the erythrocyte membrane, which is essential for normal erythrocyte function. Pesticide exposure has been linked to changes in these enzymes (MDA and GSH) that are involved in antioxidant defense mechanisms. Due to their exposure to OP insecticides, these enzymes efficiently scavenge harmful FRs and defend against lipid peroxidation [20].

MDA is a key indicator of endogenous DNA damage due to its role in the endogenous production of DNA adducts during intracellular oxidative stress. One effective method to forecast levels of oxidative stress is to measure MDA concentrations in blood plasma and tissue homogenates. The production of MDA is linked to pesticide exposure. Human erythrocyte solutions exposed to trichlorfon dose-dependently showed increased MDA levels [21].

## Materials and methods

This case-control study was carried out over a period of 1.5 years (May 2018 to November 2019) in the Department of Physiology, in collaboration with the Department of Clinical Hematology and the Center for Advanced Research (CFAR) at King George's Medical University (KGMU), Lucknow, North India. The study was conducted only after receiving clearance from the Institutional Ethics Committee at King George's Medical University, Lucknow. Ninety participants were chosen for the study from the outpatient department of Clinical Hematology, of whom 45 were cases of AA as per the diagnostic criteria of the International Agranulocytosis and Aplastic Anemia Study [22]. Forty-five subjects were age and sex-matched apparently healthy controls, preferably close relatives of patients from the same geographical area. Written informed consent was obtained from each subject before the study commenced. Patients who were unwilling to grant consent, those using medication to treat AA, those genetically predisposed to AA, with characteristics including granuloma and dysplasia of bone marrow, any other systemic illness, or subjects having a history of smoking, drinking, or using tobacco in any manner were excluded from the study.

Five millilitres of venous blood samples from each subject were collected. The blood samples were whirled in a centrifuge for 15 minutes at a speed of about 3000 rpm. To prevent haemolysis, plasma was carefully collected and kept in aliquot tubes at -80°C in a deep freezer until it was needed. Three milliliters of blood were collected in pre-heparinized vials free of pesticides as coded samples and were transported to the Center for Advanced Research for pesticide analysis by gas chromatography-tandem mass spectrometry (GC-MS/MS). Organochlorine pesticide analysis was performed using the mini QuEChERS methodology [23]. For hematological and biochemical estimation, the remaining two milliliters of blood samples were utilized.

Reagents and solutions

Each reagent and solvent used in this investigation was of analytical grade. The following were procured: acetonitrile and ethyl acetate (Reliable Scientific, Lucknow, India); n-hexane, acetone, and acetic acid (Rankem); magnesium sulfate (MgSO_4_) and sodium chloride (Sigma-Aldrich, Bangalore, India); primary-secondary amine (PSA), and C18 solid phases (Agilent Technologies); standards for twenty organochlorine pesticides (Sigma Aldrich). The standard includes 20 OCPs: alpha-hexachlorocyclohexane (HCH), beta-HCH, lindane, delta-HCH, heptachlor, aldrin, heptachlor epoxide, endosulfan peak 1, chlordane alpha cis, chlordane gamma trans, p,p’- DDE, dieldrin, endrin, endosulfan peak 2, p,p’- DDD, endrin aldehyde, p,p’- DDT, endosulfan sulphate, endrin ketone, and methoxychlor.

Extraction and clean-up of pesticide residues

The established protocol was followed. One milliliter of plasma was collected in a 15-milliliter polypropylene centrifuge tube. Then, 3 mL of acidified, 2% ethyl acetate was added and mixed. Concentrated acetic acid was used to acidify ethyl acetate; to this 0.4 gram of MgSO_4_ was added. The tube was centrifuged for 10 minutes at 6000 rpm after shaking it for 5 minutes at 50 rpm using a Rotospin rotary mixer. A 3-mL sample of the organic layer was separated and dried using a Turbovap Nitrogen Flow Evaporator. The residue was then reconstituted in 1 mL of ethyl acetate and combined with 50 milligrams of PSA to clean the sample. Utilizing a Rotospin rotary, the mixture was shaken for 5 minutes at 50 rpm and then centrifuged for 10 minutes at 8000 rpm. The remainder was reconstituted with 100 µL of ethyl acetate, and 2 µL was injected into the GC-MS/MS instrument for further analysis [23].

Estimation of oxidative stress biomarker - plasma malondialdehyde (MDA)

The lipid peroxide content was calculated using a modified technique developed by Ohkawa et al. in 1979 [24].

Principle

Acetic acid releases the tissue's lipid and protein. Lipid peroxides, hydroperoxides, and oxygen-labile double bonds interact with thiobarbituric acid (TBA) to produce color adducts, which have an absorbance maximum at 532 nm. Proteins are dissolved in the reaction mixture using sodium dodecyl sulfate (SDS).

Reagents

As listed in Table 1, SDS is prepared at 8.0% by dissolving 8.0 grams of SDS in 100 ml of double-distilled water. Acetic acid (20.0%) solution is made by diluting glacial acetic acid accordingly. β-TBA (0.8%) involves suspending 800 mg of TBA in 20 ml of double-distilled water and adjusting the pH to 7.0 using 0.1 N NaOH. This is then dissolved, and the volume is adjusted to 100 ml with double-distilled water, along with N-Butanol.

**Table 1:** Procedure of estimation of oxidative stress biomarker - plasma malondialdehyde (MDA).

Reagent	Test (µl)	Ref (µl)	Blank (µl)
Thiobarbituric acid (TBA)	750	0	750
Trichloroacetic acid (TCA)	500	500	500
Sodium dodecyl sulfate (SDS)	250	250	250
Acetic Acid	250	250	250
Double distilled water	---	750	100
Sample	100	100	--------

Procedure

The mixture is incubated for 60 minutes in a boiling water bath. After the reaction mixture is allowed to cool, 3 ml of N-butanol is added. It is then centrifuged at 4000 rpm for 15 minutes. Post-centrifugation, a clear butanol fraction is obtained, which is used to measure the absorbance at 532 nm against a blank that contains double distilled water instead of plasma.

Calculation

The quantity of MDA in the samples is determined using the molar extinction coefficient ε532 = 1.56×10^5^ M^-1^ cm^-1^ of MDA at 532 nm wavelength, with results expressed as nmol of MDA/ml plasma.

Statistical analysis

In the first step of data analysis, categorical data were presented as frequency and percentage, while continuous and normally distributed variables were presented as Mean ± SD. The Kolmogorov-Smirnov test was used to determine the normality of the data. Non-normal data were presented as Median with IQR. Comparisons between normally distributed data were performed using the independent t-test or ANOVA. The Mann-Whitney U and Kruskal-Wallis tests were used for comparisons of non-normal data. Correlations between two variables were assessed using Spearman's Rank Correlation. Data analysis was conducted using SPSS Software version 28.0 (IBM Corp. Armonk, NY, USA). Statistical significance was established at a two-tailed p-value of 0.05.

## Results

Table 2 shows a significant increase in blood plasma levels of Alpha-hexachlorocyclohexane (HCH), Delta-HCH, Heptachlor, and Aldrin according to the severity of AA.

**Table 2:** Organochlorine pesticide levels (ppb) in blood samples of cases according to severity of aplastic anemia. *p-value < 0.05 considered to be significant; HCH: Hexachlorocyclohexane.

Variables	Non-Severe AA	Severe AA	Very Severe AA	Test Value	P-value
Alpha HCH (ppb)	0.38 (0.36, 0.58)	0.39 (0.35, 0.44)	0.49 (0.41, 0.53)	6.450	0.040*
Beta HCH (ppb)	1.51 (0.57, 2.43)	0.53 (0.35, 1.2)	1.39 (0.71, 5.56)	2.453	0.293
Lindane (ppb)	0.37 (0.36, 0.93)	0.44 (0.39, 0.5)	0.53 (0.43, 0.59)	4.292	0.117
Delta HCH (ppb)	1.09 (0.89, 1.23)	1.28 (1.12, 1.43)	1.17 (1.16, 1.24)	5.935	0.051*
Heptachlor (ppb)	0.52 (0.08, 0.53)	1.35 (0.65, 1.74)	2.28 (1.78, 2.72)	10.275	0.006*
Aldrin (ppb)	41.82 (1.38, 62.83)	58.35 (55.43, 62.27)	71.69 (63.6, 72.36)	20.838	0.000*
Heptachlor epoxide (ppb)	0	0	0	-	-
Chlordane-alpha cis (ppb)	0	0	0	-	-
Endosulphan peak 1 (ppb)	0	0	0	-	-
Chlordanegamma trans (ppb)	0	0	0	-	-

Table 3 shows a significant increase in blood plasma levels of p,p'-Dichlorodiphenyldichloroethane, Endosulfan sulfate, and Methoxychlor according to the severity of AA.

**Table 3:** Organochlorine pesticide levels (ppb) in blood samples of cases according to severity of aplastic anemia. *p-value <0.05 considered to be significant. p,p’-DDE: p,p'-Dichlorodiphenyl dichloroethylene; p,p’-DDD: p,p'-Dichlorodiphenyl dichloroethane; p,p’-DDT: p,p’-Dichlorodiphenyl trichloroethane.

Variables	Non-Severe AA	Severe AA	Very Severe AA	Test Value	P-value
p,p’-DDE (ppb)	6.17 (3.99, 20.75)	5.41 (2.44, 45.16)	9.59 (6.93, 13.83)	0.933	0.627
Dieldrin (ppb)	0	0	0	-	-
Endrin (ppb)	0	0	0	-	-
Endosulfan peak 2 (ppb)	0	0	0	-	-
p, p’ - DDD (ppb)	0.12 (0.12, 1.25)	1.05 (0.71, 1.74)	1.71 (0.4, 3.8)	11.293	0.004*
EndrinAldehyde (ppb)	0.17 (0.17, 0.17)	0.17 (0.17, 0.17)	0.17 (0.17, 0.17)	0.680	0.712
p, p’ - DDT (ppb)	3.35 (1, 7.93)	4.8 (2.09, 17.22)	9.12 (2.63, 17.96)	0.877	0.645
Endosulfansulfate (ppb)	1.05 (1.05, 1.05)	1.05 (1.05, 1.05)	1.25 (1.05, 1.48)	9.263	0.010*
Endrin Ketone (ppb)	0	0	0	-	-
Methoxychlor (ppb)	0.91 (0.91, 0.91)	0.91 (0.91, 0.91)	2.35 (0.91, 4.72)	13.291	0.001*

Table 4 indicates that MDA levels were highest in cases of very severe AA, followed by severe AA, and lowest in non-severe AA.

**Table 4:** MDA levels (nmol/ml) in blood samples of aplastic anemia cases according to severity. ***p value < 0.001 MDA: Malondialdehyde

Characteristics	Non-Severe AA (n = 29)	Severe AA (n = 12)	Very Severe AA (n = 4)	F value / KW test	P-value
Malondialdehyde level (nmol / ml)	3.84 (1.44, 6.24)	4.48 (4.48, 4.48)	6.41 (1.92, 15.06)	26.457	0.000***

## Discussion

In our study, there was a significant increase in the plasma level of MDA with the severity of AA in the case group, while no significant difference was observed between the plasma levels of MDA in AA cases and controls. This suggests that oxidative stress may be related to the severity of AA.

When lipids and free radicals combine, a highly damaging chain reaction known as lipid peroxidation can occur. Polyunsaturated fatty acids (PUFA), commonly found in cell membranes, are damaged by free radicals. Unstable lipid peroxides are produced by polyunsaturated fatty acids. These quickly break down into several chemicals, including MDA, the main metabolite of arachidonic acid.

Reactive oxygen species accumulation in the bone marrow stroma, as observed in a study on aging mice, suggests that oxidative stress reduces bone marrow cellularity by altering the microenvironment [25]. Our findings are supported by observations that exposure to pesticides is associated with MDA formation. Human erythrocyte solutions treated with trichlorfon exhibited a dose-dependent increase in MDA levels [21]. Similarly, a study of the occupational health risks among male agricultural workers in the mango belt near Lucknow provided strong evidence that the population exposed to pesticides had higher serum levels of lipid peroxides like MDA compared to controls [20].

Researchers observed that greater MDA levels in OP applicators compared to farmers imply enhanced lipid peroxidation. The risk of oxidative damage from oxidative stress increases with MDA levels [19]. The level of MDA and TNF alfa in the pesticide-exposed workers was significantly higher than in controls [26]. In studies of farmers exposed to pesticides and their effects on oxidative stress biomarkers and pesticide metabolites are related [27]. Pesticide exposure (plasma organochlorine levels) in our study may act as a stressor which may initiate a response of alarmin of oxidative stress/lipid peroxidation (MDA) from damaged tissue and then could have led to suppression of hematopoiesis which could be a possible factor for the development of AA. Nonetheless, population-based investigations and prospective large follow-up studies are necessary due to the limitations of our study.

## Conclusions

In this study, MDA levels (oxidative stress) may be linked to the severity of AA. MDA levels were found to be highest in cases of very severe aplastic anemia (VSAA), followed by severe aplastic anemia (SAA), and lowest in non-severe aplastic anemia (NSAA). Pesticide exposure (plasma organochlorine levels) in our study could act as a stressor, potentially initiating a response of alarmin from oxidative stress or lipid peroxidation (MDA) from damaged tissue, which could then lead to the suppression of hematopoiesis, a possible factor in the development of aplastic anemia. In this regard, our findings support the need for population-based investigations and prospective follow-up studies for further evaluation.
